# Healthy tissue metabolism assessed by [^18^F]FDG PET/CT as a marker of prognosis and adverse events in advanced Hodgkin lymphoma patients

**DOI:** 10.1038/s41598-024-63349-5

**Published:** 2024-06-01

**Authors:** Afnan A. Malaih, Amy A. Kirkwood, Peter Johnson, Vivek Radhakrishnan, Barbara M. Fischer, Sally F. Barrington

**Affiliations:** 1 King’s College London and Guy’s and St Thomas’ PET Centre, School of Biomedical Engineering and Imaging Sciences, King’s College London, Kings Health Partners, London, UK; 2 Radiologic Sciences, Faculty of Applied Medical Sciences, King Abdulaziz University, Jeddah, Saudi Arabia; 3 Cancer Research UK and UCL Cancer Trials Centre, UCL Cancer Institute, University College London, London, UK; 4 Cancer Research UK Centre, University of Southampton, Southampton, UK; 5 Cancer Care Group, University Hospital of Southampton, Southampton, UK; 6 Department of Clinical Physiology and Nuclear Medicine, Rigshospitalet, University of Copenhagen, Copenhagen, Denmark

**Keywords:** Lymphoma, Prognostic markers

## Abstract

The aim of the study was to assess healthy tissue metabolism (HTM) using 2-deoxy-2-[^18^F]fluoro-D-glucose ([^18^F]FDG) positron emission tomography/computed tomography (PET/CT) during chemotherapy in Hodgkin lymphoma (HL) and the association of HTM with baseline metabolic tumour volume (MTV), haematological parameters, adverse events (AEs), early response and progression-free survival (PFS). We retrospectively identified 200 patients with advanced HL from the RATHL trial with [^18^F]FDG-PET/CT before (PET0) and following 2 cycles of chemotherapy (PET2). [^18^F]FDG-uptake was measured in bone marrow (BM), spleen, liver and mediastinal blood pool (MBP). Deauville score (DS) 1–3 was used to classify responders and DS 4–5, non-responders. [^18^F]FDG-uptake decreased significantly in BM and spleen and increased in liver and MBP at PET2 (all *p* < 0.0001), but was not associated with MTV. Higher BM uptake at PET0 was associated with lower baseline haemoglobin and higher absolute neutrophil counts, platelets, and white blood cells. High BM, spleen, and liver uptake at PET0 was associated with neutropenia after cycles 1–2. BM uptake at PET0 was associated with treatment failure at PET2 and non-responders with higher BM uptake at PET2 had significantly inferior PFS (*p* = 0.023; hazard ratio = 2.31). Based on these results, we concluded that the change in HTM during chemotherapy was most likely a direct impact of chemotherapy rather than a change in MTV. BM uptake has prognostic value in HL.

## Introduction

2-deoxy-2-[^18^F]fluoro-D-glucose ([^18^F]FDG) positron emission tomography/computed tomography (PET/CT) is used for staging and response assessment of patients with Hodgkin lymphoma (HL)^1^. Various quantitative PET metrics have been explored for the assessment of prognosis, including the baseline metabolic tumour volume (MTV), total lesion glycolysis (TLG)^2–4^ and semi-quantitative assessment using standardised uptake values (SUV) to confirm visual assessment for response evaluation of lymphoma lesions compared with reference tissues in the Deauville score (DS)^5^. Recently, assessment of metabolism in tissues devoid of cancer in patients with lymphoma and solid cancers has been studied to determine if healthy tissue metabolism (HTM) can provide prognostic information and predict adverse events (AEs)^6–9^.

Repeatability studies have indicated that [^18^F]FDG-uptake in the liver and mediastinal blood pool (MBP) are quite stable over time^10–12^; consequently, these tissues have been favoured as reference backgrounds for qualitative and semi-quantitative PET assessment of lymphoma^1^. However, liver and MBP uptake can change during chemotherapy, suggesting treatment may affect uptake in these reference tissues^13,14^. An inverse relationship has been reported for baseline liver and MBP uptake with pretreatment MTV and TLG^14^. It has been postulated there may be a ‘sink effect’ underlying this relationship, whereby less radiotracer is available for other tissues in the presence of large tumour volumes^15^. Whether change in liver and MBP uptake is primarily related to tumour uptake or other factors is not clearly understood.

[^18^F]FDG-uptake in lymphoid tissues such as bone marrow (BM) and spleen has been reported to be associated with pretreatment haematological parameters, e.g. reduced haemoglobin (Hb), raised white blood cell (WBC) and absolute neutrophil count (ANC)^16–19^. Both anaemia and leucocytosis are adverse risk factors in the international prognostic score (IPS) used in HL^20^. Furthermore, the prognostic value of [^18^F]FDG-uptake in BM involved by lymphoma has been demonstrated in several studies^21,22^. Reports also suggest that spleen metabolism can be associated with poor prognosis or prolonged clinical benefit in oncologic patients receiving different treatment regimens^23–25^.

This study was designed to assess (i) changes of [^18^F]FDG-uptake in liver, MBP, BM and spleen in patients with newly diagnosed HL from the Response Adapted Therapy in Advanced Hodgkin Lymphoma (RATHL) trial, (ii) whether these changes were associated with MTV and haematological parameters, (iii) whether HTM could predict AEs, and (iv) whether HTM was associated with early treatment response and patient outcome.

## Methods

### Study design and subjects

All patients enrolled in the RATHL study gave written informed consent prior to trial entry following the principles of the Declaration of Helsinki^26,27^. This retrospective work was approved by the ethics committee (South Central—Hampshire B Research Ethics Committee reference 08/H0504/15).

The inclusion criteria comprised patients who were recruited in the multicentre randomised phase III RATHL trial between 2008 and 2012. The trial was conducted in compliance with the protocol as previously reported for patients with newly diagnosed advanced classical HL, who were ≥ 18 years old with Ann Arbor stage IIB to IV or stage IIA with adverse features^26,27^. Patients had no prior cancer treatment with adequate BM function unless affected by lymphoma^26^. Patients with history of active malignancy or contraindications to chemotherapy were excluded^26^.

All patients received 2 cycles of ABVD (doxorubicin, bleomycin, vinblastine, dacarbazine) and underwent [^18^F]FDG-PET/CT scans at baseline (PET0) and after 2 cycles of treatment (PET2)^26,27^. According to the RATHL protocol all PET2 ‘negative’ patients were randomised 1:1 to continue 4 cycles of ABVD or AVD (doxorubicin, vinblastine, dacarbazine)^26,27^. PET2 ‘positive’ patients received escalated BEACOPP (bleomycin, etoposide, doxorubicin, cyclophosphamide, vincristine, procarbazine and prednisone) or BEACOPP-14 according to centre choice^26,27^.

### Imaging

[^18^F]FDG-PET/CT scans analysed in this study were acquired at multiple centres using standardised procedures for imaging acquisition and quality control according to the RATHL trial protocol^26,27^. In short, patients underwent a baseline [^18^F]FDG-PET/CT scan within 28 days prior to study registration and a second scan within 9 to 13 days following day 15 of cycle 2 of ABVD^26,27^. Patients were instructed to fast for at least 4 h prior to the scan and measured blood glucose level did not exceed 11 mmol/L for all patients. Image acquisition was performed within 60 ± 10 min after intravenous administration of 350 to 550 MBq of [^18^F]FDG^26,27^.

### Image analyses

[^18^F]FDG-PET/CT scans were analysed using a custom-made workflow in MIM software, version 7.1.5 (MIM software Inc., Cleveland, Ohio) by a PET technologist under the supervision of a PET physician (with > 10 years’ experience). Both readers were blinded to patient treatment and outcome. HTM was measured in BM, spleen, liver and MBP at PET0 and PET2 and the change in HTM was calculated. BM uptake was measured using a 2 cm spherical volume of interest (VOI) placed in T8 and L3 (or an adjacent vertebral body if involved by lymphoma). The mean SUVs from both vertebrae were averaged. In the spleen, [^18^F]FDG-uptake was measured using a 3 cm VOI. In the liver, a 6 cm VOI was placed in the right lobe^13^. In both organs care was taken to avoid including spill-over activity from adjacent tissues as well as avoiding areas of focal abnormal uptake. Where extensive disease involvement precluded measurement of normal tissue, the organ was excluded from the analysis. MBP was assessed using a 1.5 cm VOI in the descending aorta avoiding the aortic wall. The mean SUV was used for analyses of HTM as we, and others, have previously reported this to be a more stable measurement than the maximum SUV^28,29^. A fixed SUV ≥ 4.0 was used as the threshold for MTV, as the easiest to apply with the highest clinical prognostic performance from 6 published methods in HL^30,31^.

The central review from the original trial was used to assess response to treatment after the second cycle of ABVD by assigning patients as responders with DS scores 1–3 classified as complete metabolic response (CMR) (PET2−) and non-responders with DS scores 4 and 5 as non-CMR (PET2+)^26,27^.

### Haematological parameters and adverse events

Full blood count was measured at baseline and included Hb (g/dL), ANC (× 10^9^/L), WBC (× 10^9^/L), platelets (× 10^9^/L) and lymphocytes (× 10^9^/L). The adverse events were classified using the Common Terminology Criteria for Adverse Events (CTCAE) v3.0 by the National Cancer Institute^32^. Grade 3–5 AEs were considered for infection and haematological events, which included anaemia, neutropenia, leukopenia, thrombocytopenia, and lymphopenia. In the RATHL trial AEs were recorded prospectively at each cycle, and 30 days post last trial treatment (serious AEs were reportable after this point if believed to be related to trial treatment)^26^. All data were analysed using data prospectively collected in the original trial at cycles 1–2 and cycles 3 +, with the latter adjusted for treatment i.e. ABVD, AVD or BEACOPP.

### Statistical analyses

This study was initially designed as a case control-study comparing patients with and without grade 3–5 infection at any timepoint, with a sample size calculated to show a difference between these groups. Due to associations between HTM and response (leading to treatment escalation), the trial could not be analysed in this fashion; however, no retrospective power calculations were performed, and all analyses were performed on the original sample of N = 200.

Associations between continuous variables were tested using linear regression, with logistic regression used to assess the associations between HTM and binary outcomes (AEs and response) and Cox regression to assess associations with progression-free survival (PFS). PFS was measured from the date of registration until the date of the first event (progression or death from any cause) with patients who were alive and progression free censored at the last date seen. The change in HTM SUVmean from PET0 to PET2 was analysed as a percentage change i.e. PET2 as a percentage of PET0, with the difference between timepoints assessed using a Wilcoxon signed rank test. For MTV analysis, patients were excluded if they had BM or spleen involvement by [^18^F]FDG-PET/CT that precluded the assessment of HTM (i.e. healthy uninvolved BM and spleen). All analyses were performed using STATA version 18.0 (STATAcorp, Texas).

## Results

### Patients

Results were available for 198/200 patients recruited between December 3, 2008 and December 20, 2012. Two patients were excluded due to large body habitus and tracer extravasation respectively, which visibly reduced whole body uptake and with likely impact on quantitative measurements. Spleen and liver measurements could not be assessed due to extensive lymphomatous involvement in 8 patients and 1 patient respectively. The baseline clinical characteristics of the study population are presented in Table 1.

**Table 1:** Patient clinical characteristics.

Characteristics	All patients N (%)
Number of patients	198
Age (years), median (IQR)	37 (26–55)
Male sex	106 (53.5)
ECOG performance status	
0	136 (68.7)
1	51 (25.8)
2	9 (4.6)
3	2 (1.0)
Ann Arbor stage	
II	88 (44.4)
III	53 (26.8)
IV	57 (28.8)
B symptoms	147 (74.2)
Bulky disease	69 (34.9)
International Prognostic Score	
≤ 2	100 (50.5)
> 2	98 (49.5)
Treatment	
ABVD	75 (37.9)
AVD	75 (37.9)
BEACOPP	46 (23.2)
PET2 + , withdrawn^1^	2 (1.0)
Bone marrow involvement*	60 (30.3)
Spleen involvement*	131 (66.2)
Baseline MTV volume (ml), median (IQR)	174.4 (66.9–365.93)

^1^Withdrew post PET2 and further treatment unknown, *Bone marrow and spleen involvement based on [^18^F]FDG-PET/CT findings. *N* (%) number of patients and percentages unless indicated within brackets, *ECOG* Eastern Cooperative Oncology Group, *IQR* Interquartile range, *ABVD* doxorubicin, bleomycin, vinblastine, dacarbazine, *AVD* doxorubicin, vinblastine, dacarbazine, *BEACOPP* bleomycin, etoposide, doxorubicin, cyclophosphamide, vincristine, procarbazine and prednisone.

### Change in HTM during chemotherapy treatment

**Figure 1:**
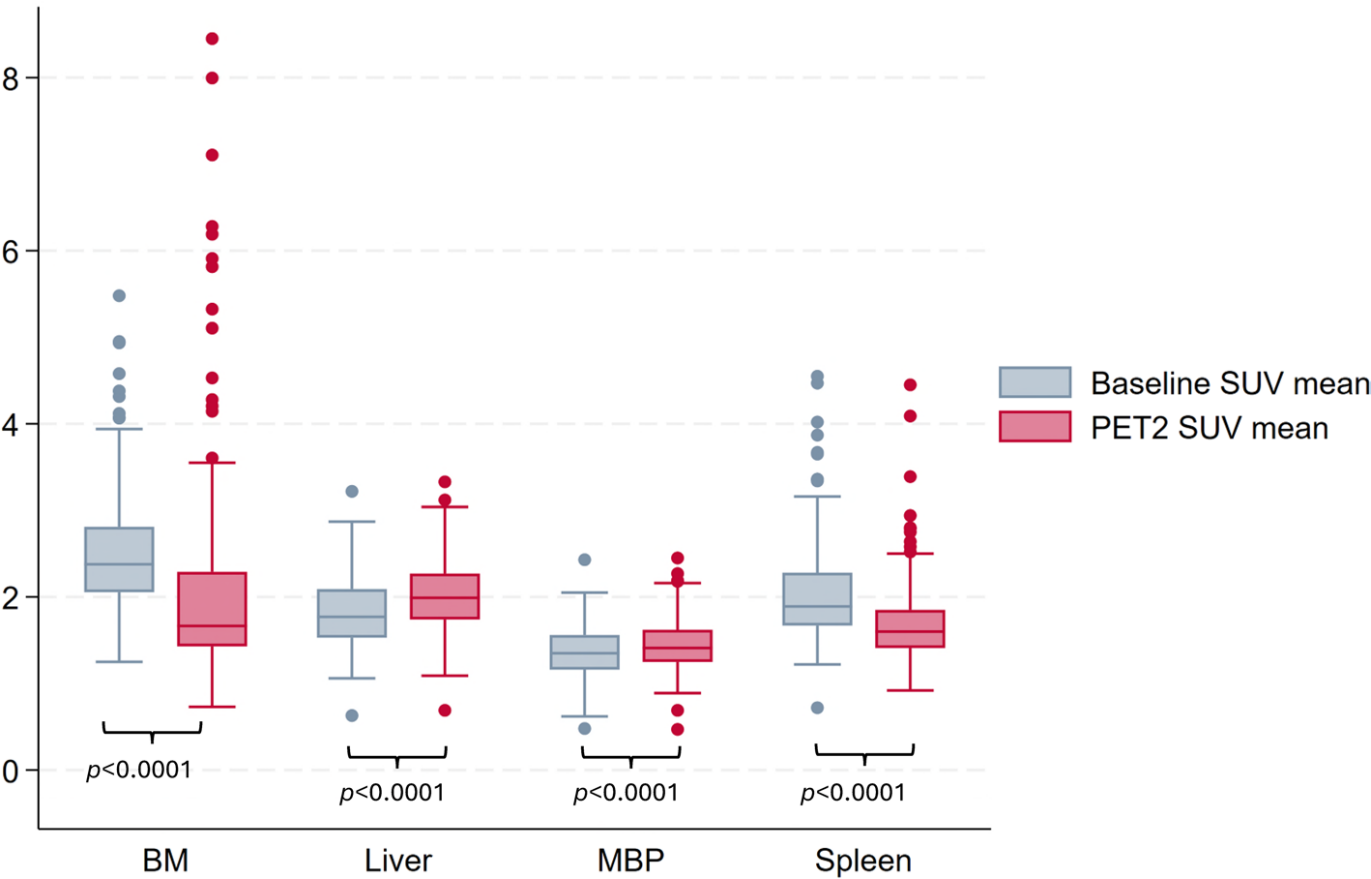
Box plot representing the percentage change in healthy tissue SUVmean between PET0 (baseline) and PET2. SUVmean in BM and spleen significantly decreased and increased significantly in liver and MBP. *BM* bone marrow, *MBP* mediastinal blood pool, *SUVmean* mean standardized uptake value.

There was a significant decrease in [^18^F]FDG-uptake in the BM and spleen and a significant increase in the liver and MBP during cycles 1–2 of chemotherapy (Fig. 1). The maximum change was seen in the BM with a small absolute median decrease in SUVmean of 0.62.

### HTM, MTV and haematological parameters

There was no significant association between baseline MTV and HTM at PET0 or PET2 nor with change in HTM.

At PET0, higher BM uptake was associated with lower baseline Hb. Conversely, higher BM uptake was associated with higher ANC, WBC, and platelet count (all *p* < 0.01). Higher liver and MBP uptake were associated with higher baseline Hb (all *p* < 0.01), and higher liver uptake was associated with lower platelet count *p* = 0.028. [^18^F]FDG-uptake in the spleen was not associated with any of the baseline haematological parameters (Supplementary Table S1).

### HTM and treatment related AEs

There was no association between HTM and the risk of infection (Table 2).

**Table 2:** Association of SUVmean in healthy tissues at PET0, PET2 and the change in SUVmean from PET0 to PET2 with adverse events (infection, neutropenia, or leukopenia) during cycles 1–2 and during cycles 3 +.

Tissue of interest	PET0 (SUVmean)			PET2 (SUVmean)			Change from PET0 to PET2 (SUVmean)^∆^		
	Adverse events (cycle 1–2)			Adverse events (cycles 3 +)					
	Events/N	OR (95% CI)	*p*-value	Events/N	OR (95% CI)^‡^	*p*-value	Events/N	OR (95% CI)^‡^	*p*-value
Infection									
BM	34/198	1.18 (0.72–1.92)	0.51	81/194	1.06 (0.83–1.36)	0.64	81/194	0.96 (0.85–1.09)	0.57
Spleen	34/198	1.57 (0.87–2.85)	0.14	79/186	1.47 (0.78–2.76)	0.24	79/186	1.02 (0.86–1.20)	0.85
Liver	34/198	1.61 (0.64–4.07)	0.31	80/193	1.37 (0.65–2.90)	0.41	80/193	1.02 (0.88–1.19)	0.76
MBP	34/198	1.07 (0.31 –3.67)	0.91	81/194	1.55 (0.54–4.41)	0.42	81/194	0.97 (0.83–1.13)	0.68
Neutropenia*									
ANC	126/197	0.97 (0.92–1.02)	0.18	128/193	0.91 (0.82–1.07)	0.07	128/193	0.91 (0.82–1.07)	0.07
BM	**126/197**	**1.79 (1.09–2.96)**	**0.02**	128/193	0.75 (0.46–1.21)^∫^	0.23	**128/193**	**0.84 (0.71–1.00)**^**∫**^	**0.04**
Spleen	**123/189**	**2.16 (1.13–4.13)**	**0.02**	124/185	0.55 (0.20–1.55)^∫^	0.26	**124/185**	**0.98 (0.96–1.00)**^**∫**^	**0.02**
Liver	**125/196**	**2.23 (1.00–4.97)**	**0.049**	128/192	1.51 (0.69–3.31)	0.31	128/192	1.00 (0.85–1.17)	> 0.99
MBP	126/197	2.45 (0.88–6.79)	0.09	128/193	1.27 (0.44–3.66)	0.66	128/193	0.97 (0.84–1.12)	0.67
Leukopenia*									
WBC	38/197	0.94 (0.87–1.01)	0.08	49/193	0.96 (0.88–1.04)	0.31	49/193	0.96 (0.88–1.04)	0.31
BM	38/197	1.53 (0.90–2.59)	0.12	**49/193**	**0.43 (0.22–0.87)**^**∫**^	**0.02**	**49/193**	**0.76 (0.61–0.93)**^**∫**^	**0.009**
Spleen	37/189	1.07 (0.58–1.96)	0.83	49/185	0.40 (0.12–1.36)^∫^	0.14	**49/185**	**0.97 (0.94–0.99)**^**∫**^	**0.01**
Liver	38/196	1.01 (0.39–2.61)	0.98	49/192	1.14 (0.35–3.79)	0.83	49/192	0.92 (0.76–1.11)	0.37
MBP	38/197	0.94 (0.28–3.12)	0.92	49/193	0.99 (0.31–3.10)	0.98	49/193	0.93 (0.78–1.11)	0.42

Significant values are in bold.

All analyses used logistic regression models treating SUVmean or the change in SUVmean (^∆^) as continuous variables. The OR at PET0 and PET2 represents the change in risk for a 1 unit increase in SUVmean. The OR for the change in SUVmean (^∆^) represents the change in risk for a 10% increase in SUVmean from PET0 to PET2. For models assessing the risk of an event in cycles 3 + (^‡^), the treatment regimen used (ABVD/AVD or BEACOPP) was included, and for those considering the risk of neutropenia and leukopenia (*), the baseline (AEs in cycle 1–2) or pre-cycle 3 (AEs in cycles 3 +) ANC or WBC (respectively) were also included as was the interaction term, where significant (^∫^). *SUVmean* mean standardised uptake value, *N* number, *OR* odds ratio, *CI* confidence interval, *BM* bone marrow, *MBP* mediastinal blood pool, *WBC* white blood cell, *ANC* absolute neutrophil count.

Higher baseline BM, spleen and liver uptake were significantly associated with neutropenia, but not leukopenia, during cycles 1–2 (Table 2). Anaemia (N = 1 and N = 2) and thrombocytopenia (N = 0 and N = 3) (during cycles 1–2 and during cycle 3 +, respectively) were rare across all cycles and therefore the association with baseline HTM could not be evaluated.

Higher BM uptake at PET2 was associated with a decreased risk of leukopenia in cycles 3 +. Similarly, when looking at the change from PET0 to PET2 those with a smaller reduction in BM and spleen uptake had a lower risk of neutropenia and leukopenia, especially in those patients with lower baseline ANC and WBC (interaction *p* for all < 0.05).

### HTM, early treatment response and patient outcome

There were 150 patients with PET2− scans (CMR) and 48 patients with PET2+ scans (non-CMR). Due to the original case/control design, the cohort was enriched with PET2+ patients (24.2%, compared to the main RATHL trial with 16.4%).

Increased BM uptake at baseline was significantly associated with failure to achieve an early response to chemotherapy. However, uptake in spleen, liver and MBP was not associated with PET2 response (Table 3).

**Table 3:** Baseline SUVmean in healthy tissue and PET2 response.

Tissue of interest	PET0 (SUVmean)		OR (95% CI)	*p*-value
	CMR (PET2−) N = 150 Median (IQR)	Non-CMR (PET2 +) N = 48 Median (IQR)		
BM	**2.3 (1.9–2.8)**	**2.6 (2.2–3.1)**	**1.64 (1.06–2.51)**	0.025*
Spleen	1.9 (1.7–2.3)	1.8 (1.7–2.3)	0.86 (0.47–1.55)	0.61
Liver	1.8 (1.5–2.1)	1.8 (1.6–2.1)	1.07 (0.47–2.46)	0.87
MBP	1.4 (1.2–1.6)	1.4 (1.2–1.6)	1.31 (0.44–3.87)	0.63

Significant values are in bold.

*Results are very similar when adjusted for age, stage, baseline haemoglobin, B-symptoms, and bulky disease (1.72, 95% CI 1.07–2.76; *p* = 0.025). *SUVmean* mean standardised uptake value, *CMR* complete metabolic response,* N* number, *BM* bone marrow, *MBP* mediastinal blood pool, *OR* odds ratio, *CI* confidence interval.

**Figure 2:**
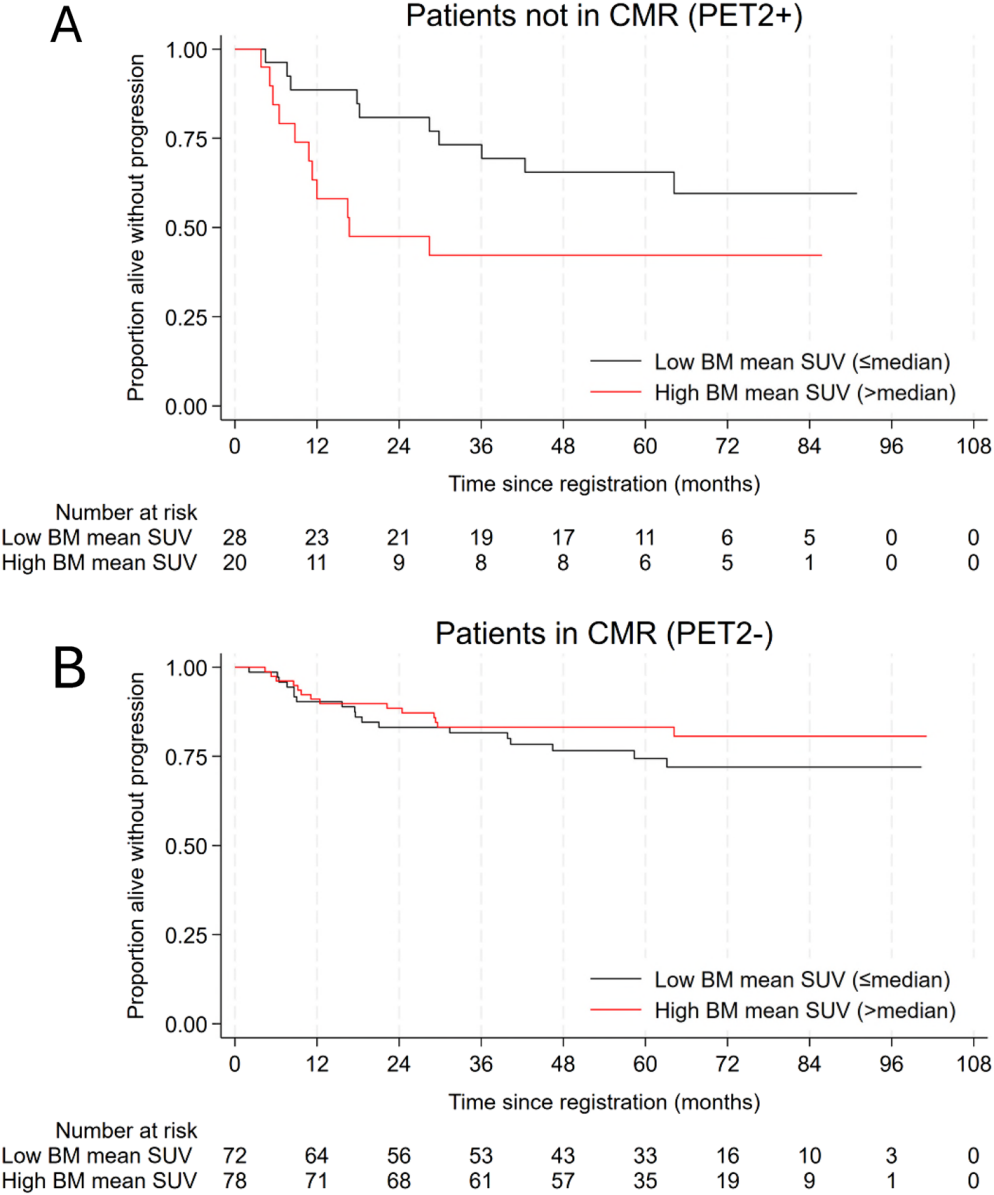
Kaplan–Meier survival curve for the association between PET2 BM SUVmean and progression-free survival in PET2+ (**A**) and PET2− (**B**) patients. BM SUVmean was split into high and low groups at the median. *BM* bone marrow, *CMR* complete metabolic response, *PET2*+ non-responders, *PET2−* responders, *SUV* standardized uptake value.

There was no association between baseline HTM (alone, or adjusted for age, stage, B-symptoms, and disease bulk) with PFS. Non-responders (PET2+) who had increased BM uptake at PET2 had significantly inferior PFS (more than double the risk of an event for a 1 unit increase in SUVmean), a finding which was not observed in responding patients (Table 4 and Fig. 2). The same pattern was seen when considering the change in BM uptake or the change in spleen uptake, though neither reached statistical significance within each group (Table 4). There was no significant association between other HTM at PET2 or change in HTM during treatment with PFS (Table 4).

**Table 4:** PET2 SUVmean and change in SUVmean in healthy tissue with PFS**.**

Tissue of interest	PET2 and PFS			Change and PFS		
	Events/N	HR (95% CI)*	*p*-value	Events/N	HR (95% CI)^**∆**^	*p*-value
BM						
PET2−	32/150	0.72 (0.48–1.08)	0.11	32/150	0.91 (0.79–1.05)	0.19
PET2+	**20/46**	**2.31 (1.12–4.75)**	**0.023**	20/46	1.18 (0.95–1.48)	0.13
Spleen						
PET2−	49/188	0.87 (0.45–1.70)	0.69	29/142	0.85 (0.71–1.02)	0.085
PET2+				20/46	1.25 (0.91–1.71)	0.16
Liver	52/195	1.25 (0.65–2.43)	0.50	52/195	1.09 (0.98–6.69)	0.09
MBP	52/196	1.32 (0.53–3.32)	0.55	52/196	1.07 (0.95–1.20)	0.25

Significant values are in bold.

SUVmean values were analysed as continuous variables (HRs shown are for a unit increase (*) or an increase of 10% from PET0 to PET2 (∆)) and were adjusted for treatment regimen (ABVD/AVD or BEACOPP) and, for analyses of change (∆), also adjusted for the PET2 SUVmean value. The interaction between the SUVmean and the response was also considered. This was significant (*p* = 0.006(*)/*p* = 0.027(**∆**) for BM and for spleen (*p* = 0.040 (∆ only)) therefore this is presented separately for PET− and PET2 + patients. *PFS* progression-free survival, *HR* hazard ratio*, N* number, *CI* confidence interval, *BM* bone marrow, *MBP* mediastinal blood pool.

## Discussion

The present study demonstrated small but significant decreases of [^18^F]FDG-uptake in the bone marrow and spleen and increases in the liver and mediastinal blood pool during chemotherapy. It is not uncommon for patients undergoing chemotherapy to show increased diffuse uptake in the bone marrow and sometimes the spleen, but overall our results showed a reduction in uptake at PET2. A potential mechanism for this observation is the myelosuppressive effect of chemotherapy on the BM^33^ and the spleen as an extramedullary site of haematopoiesis^34^. Increased liver activity at PET2, although not MBP activity, has been reported previously in HL and diffuse large B-cell lymphoma^13,35,36^. The increase in liver uptake returned to baseline values following six cycles of ABVD, suggesting it may be due to reversible changes in the liver during chemotherapy^13^. Functional changes may be linked to morphological changes reported with chemotherapy induced liver injury such as steatosis and microvascular injury^37^. The latter has been described accompanied by increased liver [^18^F]FDG-uptake in selected patients with either sinusoidal obstruction on histology or radiological changes visible on magnetic resonance imaging^38^. It is conceivable that milder changes might account for a small absolute increase in liver uptake. Another possible hypothesis suggested for the change in HTM during chemotherapy is a redistribution of [^18^F]FDG-uptake from tumour to other tissues at PET2^39^ but we and others were unable to demonstrate this as a contributory factor^35^.

Higher BM uptake was associated with lower baseline Hb and with higher ANC, WBC, and platelets. High BM uptake is independently associated with C-reactive protein (CRP) levels in patients with HL^40,41^ and also other serum inflammatory markers such as albumin, neutrophils to lymphocytes ratio, platelets to lymphocytes ratio and WBC in solid malignancies^18,42^ supportive of an inflammatory aetiology, which is well recognised in HL-associated anaemia^43^. Adams et al. however argued in their study of 21 HL patients with BM biopsies that because high BM uptake associated with anaemia was accompanied by increased cell density without increased myeloid/erythroid ratio nor activation of B and T lymphocytes, BM hyperplasia was a more likely cause^16^. They attributed elevated CRP levels in their population to increased production by the liver secondary to stimulation of cytokines such as IL-6^16^, which is linked to HL-related anaemia^43^. The upregulation of inflammatory cytokines and the increased production of CRP associated with HL is likely a contributing factor to the changes we observed in blood counts.

A strong correlation was reported in another study, similar to our findings, between higher BM uptake and higher WBC and neutrophil counts in patients with no BM involvement scanned prior to treatment^19^. This relationship was attributed to the relative abundance of neutrophils in the BM compared to other cells and their comparatively short life span, thereby reflecting acute changes in the BM at time of presentation^19^. Support for this is further suggested by the increase in BM uptake associated with administration of granulocyte colony stimulating factor and interleukin-3^19^. Cancer-related thrombocytosis is also associated with anaemia and inflammatory markers^44^ and may explain the positive association between BM uptake and platelet count.

The relationship between HTM and haematological AEs following chemotherapy is not well-understood. Our study demonstrated an association between baseline BM, spleen and liver uptake with neutropenia at cycles 1–2. A prospective study in 266 patients with haematological malignancy reported reduced baseline Hb to be one factor associated with febrile neutropenia in the first cycle of treatment which may be part of the same causality as increased baseline BM uptake, which was associated with neutropenia in our study even when adjusted for baseline Hb^45^. Conversely, patients with increased interim BM uptake (PET2) had a lower risk of leukopenia and neutropenia in cycle 3 onwards, perhaps because these patients experienced less myelosuppression than patients with lower BM uptake at PET2.

The association of high BM uptake with failure to achieve early response is perhaps not surprising given the association of anaemia and leucocytosis which have both been recognised as adverse prognostic factors in the IPS used for baseline prognostication in HL since 1998^20^, though in our dataset we saw that BM uptake remained significant even when adjusted for baseline Hb. Inferior PFS has been reported by others to be associated with increased baseline BM uptake in patients with HL and non-Hodgkin lymphoma^41^. In non-responding patients, increased BM uptake remained an adverse factor with patients experiencing inferior PFS in our study. This was not seen in patients with CMR who have an excellent overall prognosis after cycle 2.

The present study was limited by the retrospective design, although data were acquired prospectively in a multicentre trial using standardised PET acquisition. We also acknowledge that multiple comparisons have been performed without alpha adjustment and that this is a hypothesis generating study, with results needing to be confirmed in separate cohorts. The overall favourable prognosis in the whole RATHL population with 3-year PFS 82.6% (95% confidence interval (CI) 80.2 to 84.7) and overall survival 95.8% (95% CI 94.4 to 96.8)^26^ may limit the power for PFS analyses. Another limitation is the relatively low incidence of grade 3–4 adverse effects and lack of data for the more common, but less clinically relevant lower grade AEs. Finally, it is important to recognise that while this study offers valuable observations regarding HTM in HL patients receiving conventional chemotherapy, with increasing interest in employing immunotherapy with PD-L1 blockade in first line treatment^46,47^, our findings will also need to be investigated in populations treated with these agents.

## Conclusion

This study demonstrated significant changes in healthy tissue metabolism on [^18^F]FDG-PET/CT scans during chemotherapy in patients with HL. The changes did not appear to be related to reduction in MTV but rather to the effect of treatment. Higher BM, spleen and liver uptake were associated with the early onset of haematological AEs of neutropenia and leukopenia.

Reduced [^18^F]FDG-uptake in uninvolved BM was a marker of myelosuppression while increased BM uptake was an indication of HL-associated anaemia, early treatment failure and inferior PFS in patients who had not responded by PET2.

### Supplementary Information

Supplementary Table S1.

## Data Availability

The datasets generated during and/or analysed during the current study are available on reasonable request from Sally F. Barrington.

## References

[1] Barrington SF (2014). Role of imaging in the staging and response assessment of lymphoma: Consensus of the international conference on malignant lymphomas imaging working group. J. Clin. Oncol..

[2] Cottereau A-S (2018). Prognostic value of baseline metabolic tumor volume in early-stage Hodgkin lymphoma in the standard arm of the H10 trial. Blood.

[3] Mikhaeel NG (2016). Combination of baseline metabolic tumour volume and early response on PET/CT improves progression-free survival prediction in DLBCL. Eur. J. Nucl. Med. Mol. Imaging.

[4] Meignan M (2016). Baseline metabolic tumor volume predicts outcome in high-tumor-burden follicular lymphoma: A pooled analysis of three multicenter studies. J. Clin. Oncol..

[5] Meignan M, Gallamini A, Haioun C (2009). Report on the first international workshop on interim-PET scan in lymphoma. Leuk. Lymphoma.

[6] Sarocchi M (2018). An increase in myocardial 18-fluorodeoxyglucose uptake is associated with left ventricular ejection fraction decline in Hodgkin lymphoma patients treated with anthracycline. J. Transl. Med..

[7] Eshghi N (2018). 18F-FDG PET/CT can predict development of thyroiditis due to immunotherapy for lung cancer. J. Nucl. Med. Technol..

[8] Niedzielski JS (2016). 18F-fluorodeoxyglucose positron emission tomography can quantify and predict esophageal injury during radiation therapy. Int. J. Radiat. Oncol. Biol. Phys..

[9] Sachpekidis C, Hassel JC, Dimitrakopoulou-Strauss A (2022). Adverse effects under immune checkpoint inhibitors on [18F]FDG PET/CT imaging. Q. J. Nucl. Med. Mol. Imaging.

[10] Weber WA (2015). Repeatability of 18F-FDG PET/CT in advanced non-small cell lung cancer: Prospective assessment in 2 multicenter trials. J. Nucl. Med..

[11] Kramer GM (2016). Repeatability of quantitative whole-body 18F-FDG PET/CT uptake measures as function of uptake interval and lesion selection in non-small cell lung cancer patients. J. Nucl. Med..

[12] Paquet N, Albert A, Foidart J, Hustinx R (2004). Within-patient variability of 18F-FDG: Standardized uptake values in normal tissues. J. Nucl. Med..

[13] Chiaravalloti A (2014). Factors affecting intrapatient liver and mediastinal blood pool 18F-FDG standardized uptake value changes during ABVD chemotherapy in Hodgkin’s lymphoma. Eur. J. Nucl. Med. Mol. Imaging.

[14] Kim SJ (2016). Intra-patient variability of FDG standardized uptake values in mediastinal blood pool, liver, and myocardium during R-CHOP chemotherapy in patients with diffuse large B-cell lymphoma. Nucl. Med. Mol. Imaging.

[15] Viglianti BL (2018). Effects of tumor burden on reference tissue standardized uptake for PET imaging: Modification of PERCIST criteria. Radiology.

[16] Adams HJA (2016). Variety in bone marrow 18F-FDG uptake in Hodgkin lymphoma patients without lymphomatous bone marrow involvement: Does it have an explanation?. Nucl. Med. Commun..

[17] Nam HY (2010). The clinical implication and prediction of diffuse splenic FDG uptake during cancer surveillance. Clin. Nucl. Med..

[18] Lee JW, Na JO, Kang DY, Lee SY, Lee SM (2017). Prognostic significance of FDG uptake of bone marrow on PET/CT in patients with non–small-cell lung cancer after curative surgical resection. Clin. Lung. Cancer.

[19] Murata Y (2006). Correlations between 18F-FDG uptake by bone marrow and hematological parameters: measurements by PET/CT. Nucl. Med. Biol..

[20] Hasenclever D, Diehl V (1998). A prognostic score for advanced Hodgkin’s disease. N. Engl. J. Med..

[21] Lee JH (2021). Prognostic significance of bone marrow and spleen 18F-FDG uptake in patients with colorectal cancer. Sci. Rep..

[22] Lee JW, Seo KH, Kim ES, Lee SM (2017). The role of 18F-fluorodeoxyglucose uptake of bone marrow on PET/CT in predicting clinical outcomes in non-small cell lung cancer patients treated with chemoradiotherapy. Eur. Radiol..

[23] Dercle L (2018). Kinetics and nadir of responses to immune checkpoint blockade by anti-PD1 in patients with classical Hodgkin lymphoma. Eur. J. Cancer.

[24] Kim SY (2019). Diffuse splenic FDG uptake is predictive of clinical outcomes in patients with rectal cancer. Sci. Rep..

[25] Wong A (2020). 18F-FDG PET/CT based spleen to liver ratio associates with clinical outcome to ipilimumab in patients with metastatic melanoma. Cancer Imaging.

[26] Johnson P (2016). Adapted treatment guided by interim PET-CT scan in advanced Hodgkin’s lymphoma. N. Engl. J. Med..

[27] Barrington SF (2016). PET-CT for staging and early response: results from the response-adapted therapy in advanced Hodgkin lymphoma study. Blood.

[28] Malaih AA (2022). Test–retest repeatability and interobserver variation of healthy tissue metabolism using 18F-FDG PET/CT of the thorax among lung cancer patients. Nucl. Med. Commun..

[29] Zwezerijnen GJC (2022). Reproducibility of [18F]FDG PET/CT liver SUV as reference or normalisation factor. Eur. J. Nucl. Med. Mol. Imaging.

[30] Barrington SF, Meignan M (2019). Time to prepare for risk adaptation in lymphoma by standardizing measurement of metabolic tumor burden. J. Nucl. Med..

[31] Driessen J (2022). The impact of semiautomatic segmentation methods on metabolic tumor volume, intensity, and dissemination radiomics in 18 F-FDG PET scans of patients with classical hodgkin lymphoma. J. Nucl. Med..

[32] National Institutes of Health, N. C. Institute. Common Terminology Criteria for Adverse Events. *Definitions*, version 3. https://ctep.cancer.gov/protocolDevelopment/electronic_applications/ctc.htm (2006).

[33] Carey PJ (2003). Drug-induced myelosuppression: Diagnosis and management. Drug Saf..

[34] Cenariu D (2021). Extramedullary hematopoiesis of the liver and spleen. J. Clin. Med..

[35] Boktor RR, Walker G, Stacey R, Gledhill S, Pitman AG (2013). Reference range for intrapatient variability in blood-pool and liver SUV for 18F-FDG PET. J. Nucl. Med..

[36] Ceriani L, Suriano S, Ruberto T, Zucca E, Giovanella L (2012). 18F-FDG uptake changes in liver and mediastinum during chemotherapy in patients with diffuse large B-cell lymphoma. Clin. Nucl. Med..

[37] Robinson PJA (2009). The effects of cancer chemotherapy on liver imaging. Eur. Radiol..

[38] Kim H (2016). Increased hepatic FDG uptake on PET/CT in hepatic sinusoidal obstructive syndrome. Oncotarget.

[39] Wu X (2017). The association between liver and tumor [18F]FDG uptake in patients with diffuse large B cell lymphoma during chemotherapy. Mol. Imaging Biol..

[40] Salaun PY (2009). Analysis of 18F-FDG PET diffuse bone marrow uptake and splenic uptake in staging of Hodgkin’s lymphoma: A reflection of disease infiltration or just inflammation?. Eur. J. Nucl. Med. Mol. Imaging.

[41] Lee JW, Lee S-C, Kim HJ, Lee SM (2017). Prognostic value of bone marrow 18F-FDG uptake on PET/CT in lymphoma patients with negative bone marrow involvement. Hell J. Nucl. Med..

[42] Lee JW, Baek MJ, Ahn TS, Lee SM (2018). Fluorine-18-fluorodeoxyglucose uptake of bone marrow on PET/CT can predict prognosis in patients with colorectal cancer after curative surgical resection. Eur. J. Gastroenterol. Hepatol..

[43] Hohaus S (2010). Anemia in Hodgkin’s lymphoma: The role of interleukin-6 and hepcidin. J. Clin. Oncol..

[44] Alexandrakis MG (2003). Levels of serum cytokines and acute phase proteins in patients with essential and cancer-related thrombocytosis. Am. J. Clin. Oncol..

[45] Moreau M (2009). A general chemotherapy myelotoxicity score to predict febrile neutropenia in hematological malignancies. Ann. Oncol..

[46] Lynch RC (2023). Concurrent pembrolizumab with AVD for untreated classical Hodgkin lymphoma. Blood.

[47] Mei M, Herrera AF (2023). The next frontier: enter PD-1 and exit PET scans?. Blood.

