# Supplementary material for: Healthy tissue metabolism assessed by [18F]FDG PET/CT as a marker of prognosis and adverse events in advanced Hodgkin lymphoma patients
Source: Sci Rep. 2024 Jun 1;14:12613. doi: 10.1038/s41598-024-63349-5 (PMC11144227; doi:10.1038/s41598-024-63349-5)
Supplement: Supplementary file 1 — Supplementary Table S1. [file 41598_2024_63349_MOESM1_ESM.pdf]

Scientific Reports

Healthy Tissue Metabolism Assessed by [^{18}F]FDG PET/CT as a Marker of Prognosis and Adverse Events in Advanced Hodgkin Lymphoma Patients

Afnan A. Malaih[1,2], Amy A. Kirkwood[3], Peter Johnson[4], Vivek Radhakrishnan[5], Barbara M. Fischer[1,6], Sally F. Barrington[1✉]

[1] The PET Imaging Centre, King's Health Partners, St. Thomas' Hospital, King's College London, London, UK. [2] Radiologic Sciences, Faculty of Applied Medical Sciences, King Abdulaziz University, Jeddah, Saudi Arabia. [3] CRUK and UCL Cancer Trials Centre, UCL Cancer Institute, University College London, London, UK. [4] Cancer Research UK Centre, University of Southampton, Southampton, UK. [5] Cancer Care Group, University Hospital of Southampton, Southampton, UK. [6] Department of Clinical Physiology and Nuclear Medicine, Rigshospitalet, University of Copenhagen, Copenhagen, Denmark. ✉email: sally.barrington@kcl.ac.uk

Tissue of interest		Coefficient (95% CI)	p-value
BM			
	Hb	**-0.53 (-0.87 to -0.19)**	**0.002**
	ANC	**3.48 (1.90 to 5.07)**	**<0.001**
	WBC	**3.45 (1.73 to 5.16)**	**<0.001**
	Platelets	**54.95 (19.90 to 90.00)**	**0.002**
	Lymphocytes	-0.022 (-0.15 to 0.10)	0.72
Spleen			
	Hb	-0.29 (-0.69 to 0.12)	0.17
	ANC	0.32 (-1.09 – 1.72)	0.66
	WBC	0.13 (-1.46 to 1.73)	0.87
	Platelets	-10.69 (-44.86 to 23.48)	0.54
	Lymphocytes	-0.08 (-0.25 to 0.10)	0.38
Liver			
	Hb	**1.21 (0.53 to 1.89)**	**0.001**
	ANC	-2.15 (-5.11 to 0.80)	0.15
	WBC	-2.54 (-5.60 to 0.52)	0.10
	Platelets	**-70.87 (-133.987 to -7.87)**	**0.028**
	Lymphocytes	-0.08 (-0.333 to 0.17)	0.54
MBP			
	Hb	**1.33 (0.42 to 2.24)**	**0.004**
	ANC	-1.08 (-3.70 to 1.53)	0.42
	WBC	-1.29 (-4.19 to 1.62)	0.38
	Platelets	-16.18 (-85.04 to 52.68)	0.64
	Lymphocytes	0.07 (-0.27 to 0.40)	0.69

Table S1. Association of healthy tissue metabolism at PET0 with baseline haematological parameters. *CI* confidence interval, *Hb* Haemoglobin, *ANC* absolute neutrophil counts, *WBC* white blood cell counts, *BM* bone marrow, *MBP*, mediastinal blood pool.
